# Deep learning model for automatic detection of incidental adrenal abnormalities on low-dose computed tomography images

**DOI:** 10.1186/s12880-026-02651-2

**Published:** 2026-08-14

**Authors:** Kexin Wang, He Wang, Shiwei Chen, Wencong Han, Wenzhi Gao, Guangyong Shi, Tong Liu, Chongchen Ma, Jiangtao Liu, Xiangpeng Wang, Zheng Zhang

**Affiliations:** 1 Department of Radiology, Peking University First Hospital, Beijing, 100034 China; 2 Department of Urology, Peking University First Hospital; Institution of Urology, Peking University; Beijing Key Laboratory of Urogenital Diseases (Male) Molecular Diagnosis and Treatment Center; National Urological Cancer Center, Beijing, 100034 China; 3 Peking University First Hospital-Miyun Hospital, Beijing, China; 4 Beijing Smart Tree Medical Technology Co., Ltd, Beijing, 102200 China

**Keywords:** Adrenal incidental abnormality, Deep learning, Automated segmentation, Automated classification, Low-dose computed tomography

## Abstract

**Background:**

To investigate the feasibility of employing deep learning models for automated segmentation and classification of adrenal incidental abnormalities on low-dose CT images.

**Methods:**

Four distinct CT cohorts were retrospectively collected for deep learning models development (cohort A, *n* = 2574; cohort B, *n* = 1205), internal evaluation (cohort C, *n* = 3681), and external evaluation (cohort D, *n* = 779). Two experienced uroradiologists independently reviewed the CT images and labeled the adrenal glands as normal or abnormal based on predefined criteria encompassing both density and morphological abnormalities, with any discrepancies resolved through consultation. The model development cohorts were divided into a training set, a validation set, and a test set. Deep learning models for segmentation and classification were trained and evaluated on internal and external sets, with the dice similarity coefficient (DSC), area under precision–recall curves (AUPRC), and area under receiver operating characteristic curves (AUROC) as evaluation metrics. Adrenal descriptions from radiology reports were extracted to compare with the model’s performance.

**Results:**

For adrenal gland segmentation, the DSC values for the test set, internal validation cohort, and external validation cohort were 0.839 (IQR: 0.783–0.871), 0.870 (IQR: 0.819–0.902), and 0.799 (IQR: 0.729–0.849), respectively. For adrenal gland classification, the AI model achieved AUPRC values of 0.913, 0.753, and 0.927 in the test set, internal validation cohort, and external validation cohort, respectively, outperforming routine radiology reporting (AUPRC: 0.809, 0.708, 0.591; all *P* < 0.05). Corresponding AUROC values were 0.956, 0.942, and 0.977 for the AI model, which also outperformed routine radiology reporting (AUROC: 0.889, 0.705, 0.551; all *P* < 0.05).

**Conclusions:**

The deep learning models showed promise in automated adrenal segmentation and classification, highlighting AI’s potential to improve detection of adrenal abnormalities in LDCT scans.

**Trial registration:**

This study has been registered on ClinicalTrials.gov on August 25, 2025, with the unique identifier NCT07198152.

## Background

Adrenal incidental abnormalities refer to a set of clinical abnormalities, e.g., adrenal incidentaloma, nodular hyperplasia, cystic lesion as well as calcification or hemorrhage, which are detected during the evaluation of diseases outside of the adrenal glands [1–3]. Currently adrenal incidental abnormalities are mostly found through routine abdominal CT and their prevalence rates range from 0.3% to 8.9%, depending on the detection protocols and image readers [4, 5]. The most common adrenal incidental abnormalities in adults are adrenal masses (adrenal incidentaloma) [4] and enlargement [5, 6]. Although the vast majority of adrenal masses are benign, up to 2–7% may be malignant or hormonally active, requiring further evaluation based on their size and characteristics [5]. Up to 13.5% of individuals are found with incidental adrenal enlargement on CT scans [6], which is associated with adrenal dysfunction and other related health risks [7–9].

The guidelines for the management of adrenal incidentalomas published by several professional societies [10–13] have emphasized that early and consistent detection of adrenal abnormalities is crucial for determining their future management and follow-up. However, the conventional Computed Tomography is not suitable for population screening due to its high radiation dose compared with low-dose chest Computed Tomography (LDCT), which has been widely applied as a routine health screening method, especially for lung cancer screening in adults. LDCT generally covers the upper abdomen so that the adrenal abnormalities can be detected during LDCT image interpretation [14].

However, the adrenal gland abnormalities are commonly neglected in reading CT scans due to the small sizes of the abnormalities [15, 16]. Deep learning models in the field of medical imaging have been applied to classify adrenal pathologies, including differentiating neuroblastomas from hematomas [17], discerning adenomas from metastases [18, 19], and diagnosing conditions, such as aldosteronism, Cushing’s syndrome, and pheochromocytomas [20]. In addition, these applications are frequently used on the patients already diagnosed with these illnesses. Robinson-Weiss et al. developed a two-stage machine learning pipeline that successfully segmented adrenal glands and differentiated normal glands from mass-containing glands on CT images of the abdomen [3]. Kusunoki M et al. developed and validated deep learning-based models for differentiating adrenal adenomas from non-adrenal adenomas utilizing abdominal multiphasic CT images [21]. Actually, the segmentation or classification performance of machine learning demonstrated non-significant difference or even better than that of the radiologists in these previous studies. In spite of these achieved progresses, available investigations are still limited in sample size or lack external validation. To date, few researches focus on the automated segmentation of the adrenal glands by deep learning models to verify their abnormalities [22]. Crucially, few studies extend to thoracic LDCT scans, or encompass the full spectrum of adrenal abnormalities.

We postulated that automated segmentation of the entire adrenal glands with no further manual intervention, coupled with automated classification, would help to identify the adrenal incidental abnormalities. Such a method may potentially decrease the neglected cases by the radiologists of the abnormalities in the adrenal glands due to the small sizes and become advantageous in the labor-intensive process of interpreting LDCT scans. Therefore, the purpose of this study is to investigate the feasibility of employing deep learning models for automated segmentation and classification of adrenal incidental abnormalities on low-dose CT images.

## Methods

### Data inclusion

This retrospective study was approved by the Institutional Review Board of Peking University First Hospital (approval number: 2022yan068-002). The requirement for informed consent was waived due to the retrospective nature of the study and the use of de-identified data. This study included four distinct cohorts. Cohort A was derived from Hospital One and consisted of thoracic LDCT scans collected from March 2018 through December 2021. To increase the sample size of patients with adrenal abnormalities, Cohort B included patients from Hospital One who underwent abdominal CT scans from October 2021 through December 2021 and whose radiological reports indicated adrenal abnormalities. Cohort B (abdominal CT) was included to enrich the training data with abnormal adrenal cases, addressing the class imbalance inherent in incidental LDCT screening. The combined cohorts were treated as a transfer-learning setting, in which abdominal CT provided supplementary abnormal examples for model development. Data from Cohorts A and B were utilized for AI model development. Cohort C, also from Hospital One, consisted of consecutive thoracic LDCT scans from July 2022 through April 2023. Cohort D, sourced from Hospital Two, employed the same inclusion criteria as Cohort C. Cohorts C and D were designated for the internal and external validation of the AI models.

**Fig. 1:**
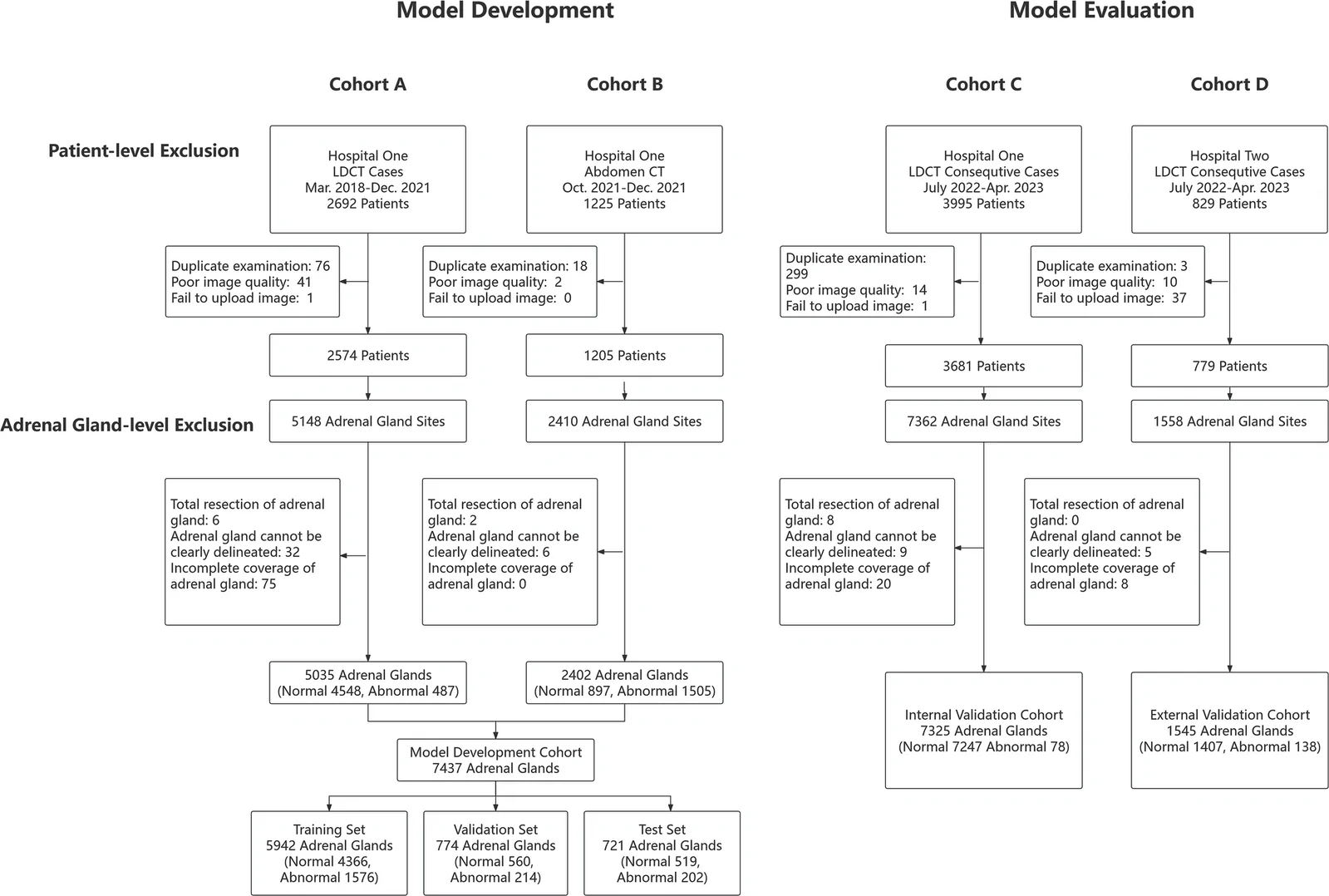
Data inclusion and model development process. This study encompassed four distinct cohorts. Cohort A (thoracic LDCT, Hospital One) and Cohort B (abdomen CT, Hospital One) served as the model development dataset. These cohorts were further divided into training, validation, and test sets to facilitate the model training process. Cohort C (thoracic LDCT, Hospital One) was collected to serve as an internal validation cohort. Cohort D (thoracic LDCT, Hospital Two) was specifically incorporated to provide external validation of the model. LDCT, low-dose computed tomography

This study employed a two-stage inclusion process to ensure data quality and analytical clarity. Stage 1: Patient-level screening. Consecutive CT examinations were screened for eligibility. Exclusion criteria were: (1) duplicate examination; (2) poor image quality precluding adequate visualization; and (3) failure to upload images. After Stage 1, 3,779, 3,681, and 779 patients’ CT examinations were retained in the model development, internal validation, and external validation cohorts, respectively. Stage 2: Gland-level annotation and inclusion. Two experienced uroradiologists independently labeled adrenal glands on the retained CT images. Glands were excluded if: (1) the adrenal gland had been totally resected; (2) the gland could not be clearly delineated from surrounding structures; or (3) adrenal glands had incomplete coverage on the CT scan. Cases with unilateral incomplete adrenal coverage were retained, with only the visible adrenal gland included in the analysis. After Stage 2, final analyzed gland counts were 7,437, 7,325, and 1,545, respectively. The detailed data flow, including per-cohort exclusion counts and reasons, is shown in Fig. 1.

### Scanning protocol

All CT examinations in Cohorts A, C, and D were performed without IV contrast administration, consistent with the standard protocol for LDCT screening. For Cohort B, all CT examinations were performed with IV contrast administration using a standardized protocol; however, only the non-contrast phase images were used in this study.

All the CT images were collected from 12 scanners manufactured by five different vendors. The model development cohorts were acquired from scanners of four vendors, and the internal validation cohort was collected from scanners of three vendors. The external validation cohort was obtained from scanners by two vendors. The tube current for the model development dataset was 133.0 (IQR: 101.0-300.0) mA, the peak tube voltage was 100 (IQR: 100–120) kV, and the scan slice thickness was 5.00 (IQR: 5.00–5.00) mm. For the internal validation cohort, the tube current was 98.0 (IQR: 77.0-120.0) mA, the peak tube voltage was 100 (IQR: 100–100) kV, and the scan slice thickness was 5.00 (IQR: 5.00–5.00) mm. The external validation cohort had a tube current of 78.0 (IQR: 65.0-92.5) mA, a peak tube voltage of 120 (IQR: 110–120) kV, and a scan slice thickness of 1.50 (IQR: 1.50–1.50) mm. All differences in scanning parameters between the model development cohorts, internal validation cohort, and external validation cohort were statistically significant (all *P* < 0.001). Details of the scanning protocols are presented in Table 1.

**Table 1:** Scanning parameters of CT imaging

	Model development dataset	Internal validation cohort	External validation cohort	*P* value
	(*N* = 3779)	(*N* = 3681)	(*N* = 779)	
**Manufacture**				
GE	732 (19.4%)	666 (18.1%)	0 (0.0%)	< 0.001
NMS	29 (0.8%)	0 (0.0%)	0 (0.0%)	
Philips	1866 (49.4%)	1930 (52.4%)	0 (0.0%)	
SIEMENS	1152 (30.5%)	1085 (29.5%)	771 (99.0%)	
UIH	0 (0.0%)	0 (0.0%)	8 (1.0%)	
**CT device**				
Discovery CT750 HD	105 (2.8%)	0 (0.0%)	0 (0.0%)	< 0.001
Emotion 16 (2007)	0 (0.0%)	0 (0.0%)	40 (5.1%)	
iCT 256	1866 (49.4%)	1930 (52.4%)	0 (0.0%)	
LightSpeed VCT	618 (16.4%)	0 (0.0%)	0 (0.0%)	
NeuViz Prime	29 (0.8%)	0 (0.0%)	0 (0.0%)	
Optima CT620	9 (0.2%)	666 (18.1%)	0 (0.0%)	
SOMATOM Definition AS+	0 (0.0%)	0 (0.0%)	527 (67.7%)	
SOMATOM Definition Flash	1113 (29.5%)	1085 (29.5%)	0 (0.0%)	
SOMATOM Drive	1 (0.0%)	0 (0.0%)	0 (0.0%)	
SOMATOM Force	38 (1.0%)	0 (0.0%)	0 (0.0%)	
SOMATOM go.Up	0 (0.0%)	0 (0.0%)	204 (26.2%)	
uCT 530	0 (0.0%)	0 (0.0%)	8 (1.0%)	
**Exam type**				
Abdomen CT	1205 (31.9%)	0 (0.0%)	0 (0.0%)	< 0.001
Chest LDCT	2574 (68.1%)	3681 (100%)	779 (100%)	
**Exposure time (ms)**				
Median (IQR)	507 (500–511)	504 (500–508)	500 (500–651)	< 0.001
**Tube current (mA)**				
Median (IQR)	133.0 (101.0-300.0)	98.0 (77.0-120)	78.0 (65.0-92.5)	< 0.001
**KVP (kV)**				
Median (IQR)	100 (100–120)	100 (100–100)	120 (110–120)	< 0.001
**Field of view (mm)**				
Median (IQR)	381 (352–416)	321 (301–346)	361 (338–382)	< 0.001
**Slice thickness (mm)**				
Median (IQR)	5.00 (5.00–5.00)	5.00 (5.00–5.00)	1.50 (1.50–1.50)	< 0.001
**Slice spacing (mm)**				
Median (IQR)	5.00 (5.00–5.00)	5.00 (5.00–5.00)	1.50 (1.50–1.50)	< 0.001
**Pixel spacing (mm)**				
Median (IQR)	0.744 (0.688–0.813)	0.627 (0.588–0.676)	0.705 (0.660–0.746)	< 0.001

### Ground truth

The adrenal gland segmentation masks were generated using a semi-automated approach to ensure both accuracy and consistency across the large dataset. Specifically, a pre-trained deep learning segmentation model [23] was first applied to all CT images to generate preliminary adrenal gland masks. Two experienced uroradiologists (with 26 and 15 years of experience, respectively) independently reviewed each preliminary segmentation mask slice-by-slice. They manually corrected any inaccuracies, including boundary adjustments, exclusion of adjacent structures (e.g., renal upper pole, liver, diaphragmatic crura), and addition of missed adrenal tissue. Discrepancies between the two radiologists were resolved through consensus consultation. The consensus-corrected masks were used as the final ground truth for model training and evaluation.

**Fig. 2:**
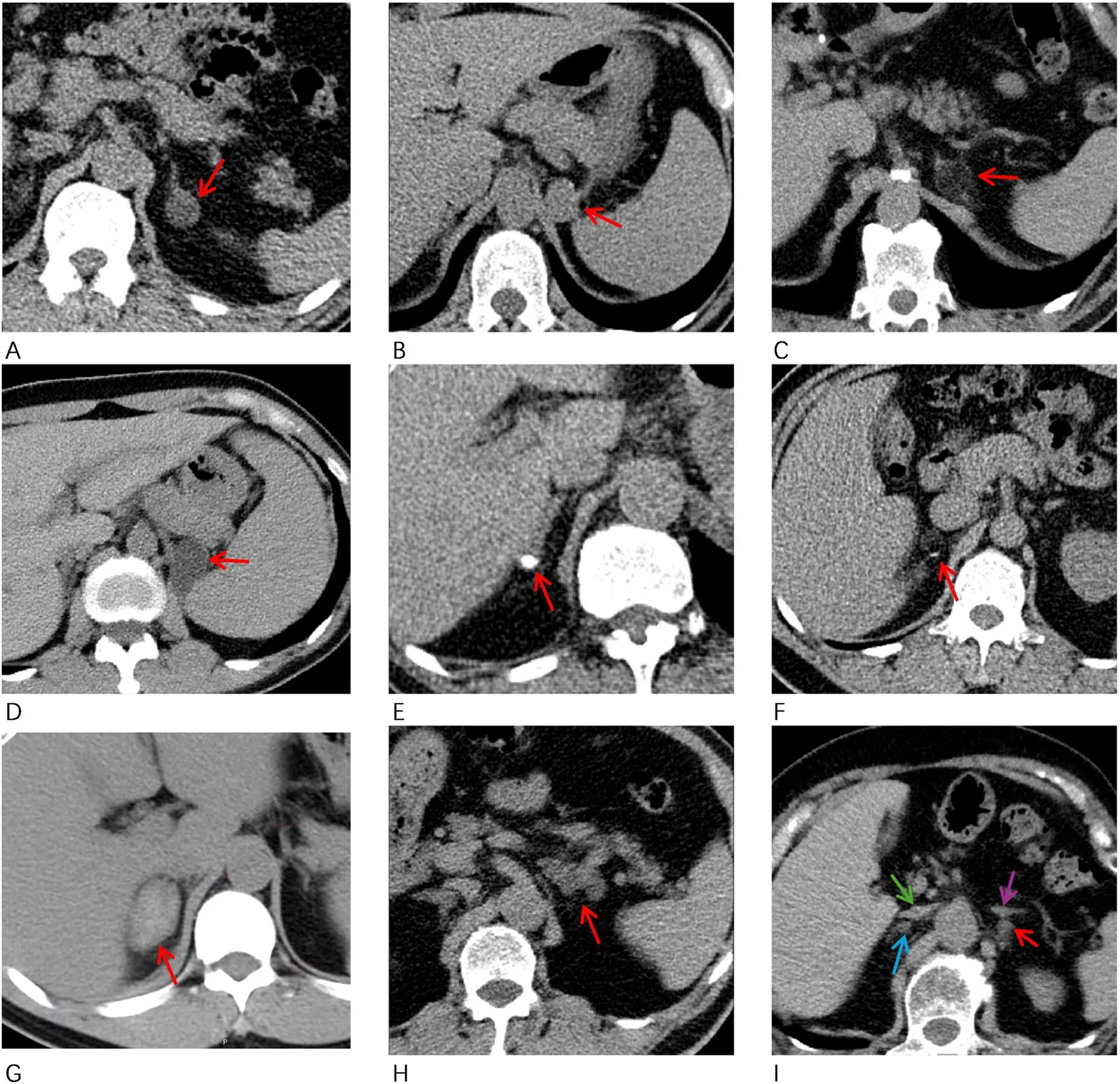
Examples of the adrenal gland abnormalities on non-contrast CT. The illustration provides examples of some adrenal gland abnormalities as seen on non-contrast CT images, with the abnormal areas in the adrenal region indicated by arrows. (**A**) Adenoma of the adrenal gland: A localized soft tissue density lesion in the adrenal region, which is roughly circular, well-defined, homogeneous, with an average CT value of 0-10 HU. (**B**) Non-specific mass or nodule in the adrenal region: A localized soft tissue lesion in the adrenal region with clear or unclear boundaries, with a CT value of 10-40 HU. (**C**) Adrenal myelolipoma: A localized fat-density lesion in the adrenal region, with a CT value ranging from -20 to -100 HU. (**D**) Cystic lesion in the adrenal region: A localized water-density lesion in the adrenal region, which is roughly circular with clear boundaries, with a CT value of approximately 0 HU. (**E**) Calcification of the adrenal gland: Single or multiple high-density lesions in the adrenal region, usually small, appearing as dots, lines, or patches, with clear boundaries, and may involve the entire adrenal gland, with a CT value of over 100 HU. (**F**) Post-operation changes in the adrenal region: The normal adrenal gland is not clearly visible, and short linear high-density objects can be seen locally, which are metal sutures or clips after surgery. (**G**) Hemorrhage in the adrenal gland: During the acute phase, it manifests as diffuse enlargement of the adrenal gland and increased density, with a CT value of 50-80 HU. (**H**) Diffuse hyperplasia of the adrenal gland: Diffuse enlargement of the adrenal gland, which can be an increase in the volume and thickness of the entire adrenal gland or an increase in the volume of the medial or lateral limbs. The boundaries are clear, with no focal abnormal density, and no formation of masses or nodules. (**I**) Nodular hyperplasia of the adrenal gland: The appearance of one or more nodules in the adrenal gland, which are iso-dense or slightly hypodense, the adrenal gland's shape is irregular, and they can occur in any part of the adrenal gland. They can be present in a single adrenal gland or bilateral adrenal glands

Two experienced uroradiologists independently reviewed the CT images to identify any adrenal gland abnormalities. Their evaluation criteria for adrenal abnormalities included the presence of either isolated or combined density and morphological abnormalities within the adrenal glands, as depicted in Fig. 2 [13, 24, 25]. Adrenal density abnormalities included conditions such as calcifications, hemorrhage, cysts, fatty infiltration, or densities that were lower than those typically found in normal adrenal tissue. Morphological abnormalities consisted of diffuse enlargement of the adrenal gland, multiple nodules, single nodules or masses, and changes suggestive of previous surgical intervention [6, 15, 26]. Any inconsistency for the cases with different identifications between the two radiologists was resolved through consultations.

### Model development

**Fig. 3:**
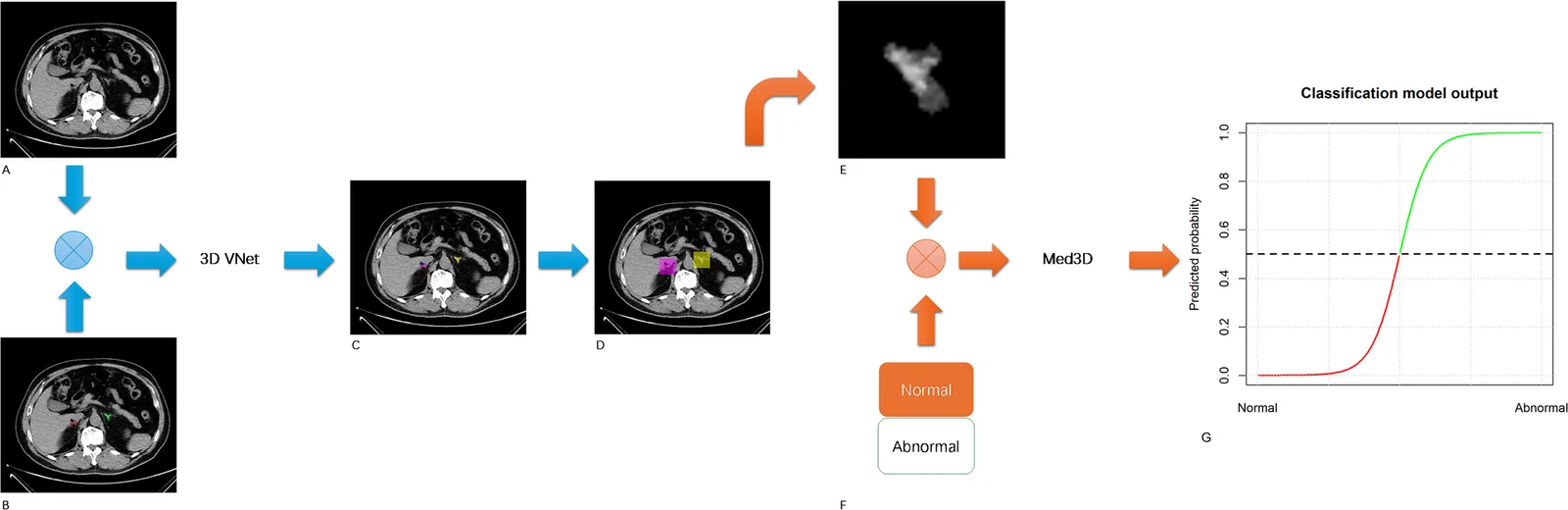
Model development process. The development of the AI model was divided into two stages. The first stage involved the segmentation of the adrenal gland, where the 3D VNet deep learning network [25, 26] was utilized. The input data consisted of complete CT images (**A**), and the bilateral adrenal regions were labeled by experts (**B**). The output was the bilateral adrenal regions predicted by the segmentation model (**C**). The second stage was the classification of the adrenal gland, which employed the Med3D deep learning model [37]. Each predicted adrenal region from the segmentation model served as a mask (**D**) to crop the CT images, yielding the adrenal bounding box (**E**), which then became the input for the Med3D model. The experts provided classifications for the adrenal gland, which were labeled either normal or abnormal (**F**). The cropped CT images and their labels were used as inputs for the Med3D model, and the output was the probability that the adrenal gland is abnormal (**G**)

The workflow of our deep learning algorithms, presented in Fig. 3, encompasses two stages: adrenal segmentation and adrenal classification.

The training processes were all performed using an NVIDIA Tesla P100 16G GPU. The algorithm was coded in Python 3.6, PyTorch 0.4.1, OpenCV 3.4.0.12, NumPy 1.16.2, and SimpleITK 1.2.0. We developed the deep learning model by using the model development dataset, which was randomly partitioned into three subsets—a training set, a validation set, and a test set—at approximate ratios of 8:1:1. To ensure the integrity of the evaluation, data from the same patient were allocated exclusively to one subset. During the training process, the training set was used for gradient descent optimization, while the validation set was employed for hyperparameter tuning and model selection. The test set was reserved for evaluating the model’s performance after training. 

In the first step, we utilized a three-dimensional deep neural network known as 3D VNet [27, 28]. This network consists of an encoder and a decoder designed to pinpoint the location of the bilateral adrenal glands. The encoder performs four down-sampling operations to construct feature maps, which are later used in the decoder through skip connections. The decoder up-samples from the latent variables and concatenates them with the preceding encoder feature maps. The input to the 3D VNet consists of the entire CT images, which undergo preprocessing before being fed into the network. To ensure uniform shape and voxel spacing across the data, the images were resampled to a fixed resolution of 256 × 192 × 96 (x × y × z). The window width was set to 300, and the window center was set to 30. Additionally, the voxel values were normalized via z score normalization. We augmented the training set data by applying random rotations (with angles up to 10°), introducing random noise, and performing parallel translations within the range of [-0.1, 0.1] pixels for both the horizontal and vertical axes. The loss function for 3D VNet was the Dice loss, which is well suited for addressing imbalanced classes that often encountered in medical imaging. The Dice coefficient *D* is defined as:

$$\:D=\frac{2\left|\mathrm{A}\bigcap\:B\right|}{\left|\mathrm{A}\right|+\left|B\right|}$$

where *A* represents the predicted segmentation, and *B* represents the ground truth. The Dice loss *L*_*Dice*_is defined as:

$$\:{L}_{Dice}=1-\frac{2{\sum}_{i}{p}_{i}{g}_{i}}{{\sum}_{i}{p}_{i}^{2}+{\sum}_{i}{g}_{i}^{2}}$$

where *p*_*i*_are the predicted probabilities for each voxel and *g*_*i*_are the ground truth labels.

In the second step, we employed a deep learning network called Med3D [29]. The segmentation backbone employed the ResNet-50 architecture, pretrained on the 3DSeg-8 dataset, with weights subsequently fine-tuned on the adrenal CT training cohorts. We retained the encoder component and replaced the decoder of the network with the convolutional and fully connected layers from the published classification network architecture. The classification convolutional layers were structured as follows: (1) a max-pooling layer with a stride of 2; (2) a convolutional layer with a kernel size of 3; (3) another max-pooling layer with a stride of 2; and (4) a second convolutional layer with a kernel size of 3. The fully connected layer of the classification network consisted of 128 neurons, which integrate and classify the image features. The output is then computed, and the classification results are presented through a Softmax function, which generated the probability distribution across classes. The input to the second stage is a bounding box derived from the adrenal masks extracted during the first step. These masks were used to crop the bounding box from the entire CT image, preparing the input for the second stage. Med3D then predicts that each adrenal gland is either normal or abnormal. The output from the second stage is a probability score indicating the likelihood of abnormality, with values ranging from 0 to 1. The loss function employed for Med3D is binary cross-entropy loss, which is defined as follows:

$$\:{L}_{BCE}=-\left[ylog\left(p\right)+\left(1-y\right)\mathrm{l}\mathrm{o}\mathrm{g}(1-p)\right]$$

where *y* is the true label (0 or 1) and *p* is the predicted probability of the instance being in class 1.

We used the Adam optimizer with a learning rate of 0.001 to update the network’s parameters. To prevent overfitting, we implemented early stopping, selecting the final model based on the criterion that the validation loss did not decrease over 50 consecutive epochs.

### Model performance assessment

We assessed the model’s performance on the test set from the model development dataset, internal validation cohort, and external validation cohort. For adrenal segmentation, we evaluated the model’s performance using the Dice score (DSC), volume similarity (VS), and average Hausdorff distance (HD) [30].

VS was calculated as:

$$\:VS=1-\frac{|{V}_{p}-{V}_{g}|}{{V}_{p}+{V}_{g}}$$

where *Vp* and *Vg* denote the volumes of the predicted segmentation and the ground-truth segmentation, respectively. VS ranges from 0 to 1, with 1 indicating identical volumes.

HD was computed as the average of all voxel-wise Hausdorff distances on the mean surface. For each boundary voxel *x* in the predicted surface (*Sp*), the minimum distance to any boundary voxel *y* in the ground-truth surface (*Sg*) was calculated. The mean of these minimum distances across all *x*∈*Sp* yields the directed mean surface distance from prediction to ground truth. The symmetric HD was then obtained by averaging this directed mean surface distance with its reverse counterpart (from ground truth to prediction):

$$\:{d}_{H}\left({S}_{p}\to\:{S}_{g}\right)=\frac{1}{\left|{S}_{p}\right|}{\sum}_{x\in\:{S}_{p}}{min}_{y\in\:{S}_{g}}\|x-y\|$$

$$\:HD=\frac{1}{2}\left[{d}_{H}\left({S}_{p}\to\:{S}_{g}\right)+{d}_{H}\left({S}_{g}\to\:{S}_{p}\right)\right]$$

This metric represents the average of all voxel-wise Hausdorff distances on the mean surface, reported in millimeters (mm).

For adrenal classification, we utilized precision–recall (PR) curves and receiver operating characteristic (ROC) curves. Given the severe class imbalance in the internal validation cohort and external validation cohort, AUPRC was prioritized as the primary headline metric, with AUROC reported secondarily. The corresponding sensitivity, positive predictive value (PPV), accuracy, specificity, and negative predictive value (NPV) were also reported. We also collected radiology reports for these patients and extracted descriptions of the adrenal glands from the reports. Both the model’s output and the radiology reports were compared against the ground truth.

### Statistical analysis

Statistical analyses were conducted using R software version 4.1.3. Continuous variables with a normal distribution are presented as mean ± standard deviation, while non-normally distributed variables are expressed as the median (interquartile range, IQR). Categorical variables are reported as frequencies and percentages. For patients’ characteristics, the Kolmogorov‒Smirnov (KS) test was used to assess the normality of the distribution, and the F test was used to assess the homogeneity of variance. The Mann‒Whitney U test or Kruskal‒Wallis test was used to compare groups with non-normally distributed data or unequal variances. For multiple comparisons, the Bonferroni method was used for adjustment. Comparisons of AUROCs were performed using the DeLong test.

The distribution of DSC values was assessed using the Anderson-Darling test, Q-Q plots, and skewness/kurtosis indices. Given the non-normal distribution of DSC, the Kruskal-Wallis H test was used for overall cohort comparisons, followed by Dunn’s test with Bonferroni adjustment for post-hoc pairwise comparisons. Epsilon-squared (ε²) was reported as the overall non-parametric effect size (negligible/small/medium/large: 0.01/0.08/0.26), and Cliff’s delta was reported for pairwise comparisons (negligible/small/medium/large: 0.147/0.330/0.474). To distinguish statistical significance from clinical relevance, a predefined threshold of |ΔDSC| ≥ 0.10 was applied to median differences; differences that were statistically significant but fell below this threshold were annotated as statistically significant but clinically negligible.

## Results

### Clinical information

The clinical characteristics of the model development, internal validation, and external validation cohorts are summarized in Table 2. The median age ranged from 47.0 to 60.0 years, and the proportion of male participants ranged from 49.2% to 65.6% across the three cohorts. The prevalence of normal adrenal glands varied substantially, from 73.3% in the development cohort to 98.9% in the internal validation cohort and 91.1% in the external validation cohort. Statistically significant differences were observed for all clinical parameters across the three groups (*P* < 0.05).

**Table 2:** Characteristics of the included data

	Model development dataset	Internal validation cohort	External validation cohort	*P* value
**Age**				
Median (IQR)	60.0 (49.0–69.0)	47.0 (37.0–60.0)	60.0 (47.0–69.0)	< 0.001
**Gender**				
F	1548 (41.6%)	1863 (50.8%)	264 (34.4%)	< 0.001
M	2171 (58.4%)	1804 (49.2%)	503 (65.6%)	
**Patient diagnosis**				
Bilateral normal	2268 (61.0%)	3602 (98.2%)	646 (83.6%)	< 0.001
Left side abnormal	759 (20.4%)	46 (1.3%)	105 (13.6%)	
Right side abnormal	217 (5.8%)	17 (0.5%)	14 (1.8%)	
Bilateral abnormal	475 (12.8%)	2 (0.1%)	8 (1.0%)	
**Adrenal diagnosis**				
Normal	5445 (73.3%)	7247 (98.9%)	1407 (91.1%)	< 0.001
Diffuse or nodular hyperplasia	689 (9.2%)	29 (0.4%)	56 (3.6%)	
Adenoma	665 (8.9%)	18 (0.2%)	31 (2.0%)	
Unspecific mass of nodule	459 (6.2%)	23 (0.3%)	46 (3.0%)	
Calcification	109 (1.5%)	7 (0.1%)	5 (0.3%)	
Myelolipoma	33 (0.4%)	0 (0.0%)	0 (0.0%)	
Hemorrhage	2 (0.0%)	0 (0.0%)	0 (0.0%)	
Post-operation change	35 (0.5%)	1 (0.0%)	0 (0.0%)	
**Left adrenal gland**				
**RL diameter (cm)**				
Median (IQR)	1.70 (1.30–2.10)	1.36 (1.08–1.68)	1.63 (1.34–2.20)	< 0.001
**AP diameter (cm)**				
Median (IQR)	2.90 (2.40–3.50)	2.58 (2.13–3.03)	3.07 (2.53–3.66)	< 0.001
**SI diameter (cm)**				
Median (IQR)	3.10 (2.60–3.90)	3.00 (2.50–3.50)	3.00 (2.50–3.45)	< 0.001
**Volume (cm^3)**				
Median (IQR)	2.37 (1.68–3.54)	1.79 (1.38–2.25)	1.95 (1.37–2.62)	< 0.001
**Right adrenal gland**				
**RL diameter (cm)**				
Median (IQR)	1.80 (1.50–2.10)	1.46 (1.20–1.76)	1.74 (1.41–2.11)	< 0.001
**AP diameter (cm)**				
Median (IQR)	3.00 (2.60–3.40)	2.71 (2.34–3.09)	2.97 (2.56–3.49)	< 0.001
**SI diameter (cm)**				
Median (IQR)	3.40 (2.90–3.80)	3.00 (3.00-3.50)	3.30 (2.85–3.60)	< 0.001
**Volume (cm^3)**				
Median (IQR)	2.45 (1.91–3.21)	1.95 (1.54–2.37)	2.27 (1.78–2.84)	< 0.001

RL: right to left. AP: anterior to posterior. SI: superior to inferior

### Results of the adrenal segmentation model

The DSC values for the test set, internal validation cohort, and external validation cohort were 0.839 (IQR: 0.783–0.871), 0.870 (IQR: 0.819–0.902), and 0.799 (IQR: 0.729–0.849), respectively. Given the non-normal distribution of DSC (Anderson-Darling test, A = 563.76, *P* < 0.001), the Kruskal-Wallis H test was used to compare DSC across cohorts, which revealed a statistically significant difference (H = 925.7, df = 2, *P* < 0.001) with a medium overall effect size (ε² = 0.10). Post-hoc Dunn’s test with Bonferroni adjustment showed that all pairwise median differences were statistically significant (all *P* < 0.001). However, applying a predefined clinical significance threshold of |ΔDSC| ≥ 0.10, none of the differences reached clinical relevance: the internal validation cohort had a marginally higher median DSC than the test set (median difference: +0.031, Cliff’s delta = − 0.260, small, *P* < 0.001); the external validation cohort had a lower median DSC than the test set (median difference: −0.040, Cliff’s delta = 0.316, small, *P* < 0.001) and the internal validation cohort (median difference: −0.071, Cliff’s delta = 0.470, medium, *P* < 0.001). These findings demonstrate that while large sample sizes enable detection of statistically significant differences, the absolute cohort-related variation in segmentation performance (maximum |median ΔDSC| = 0.071) remained below the predefined clinical significance threshold.

**Fig. 4:**
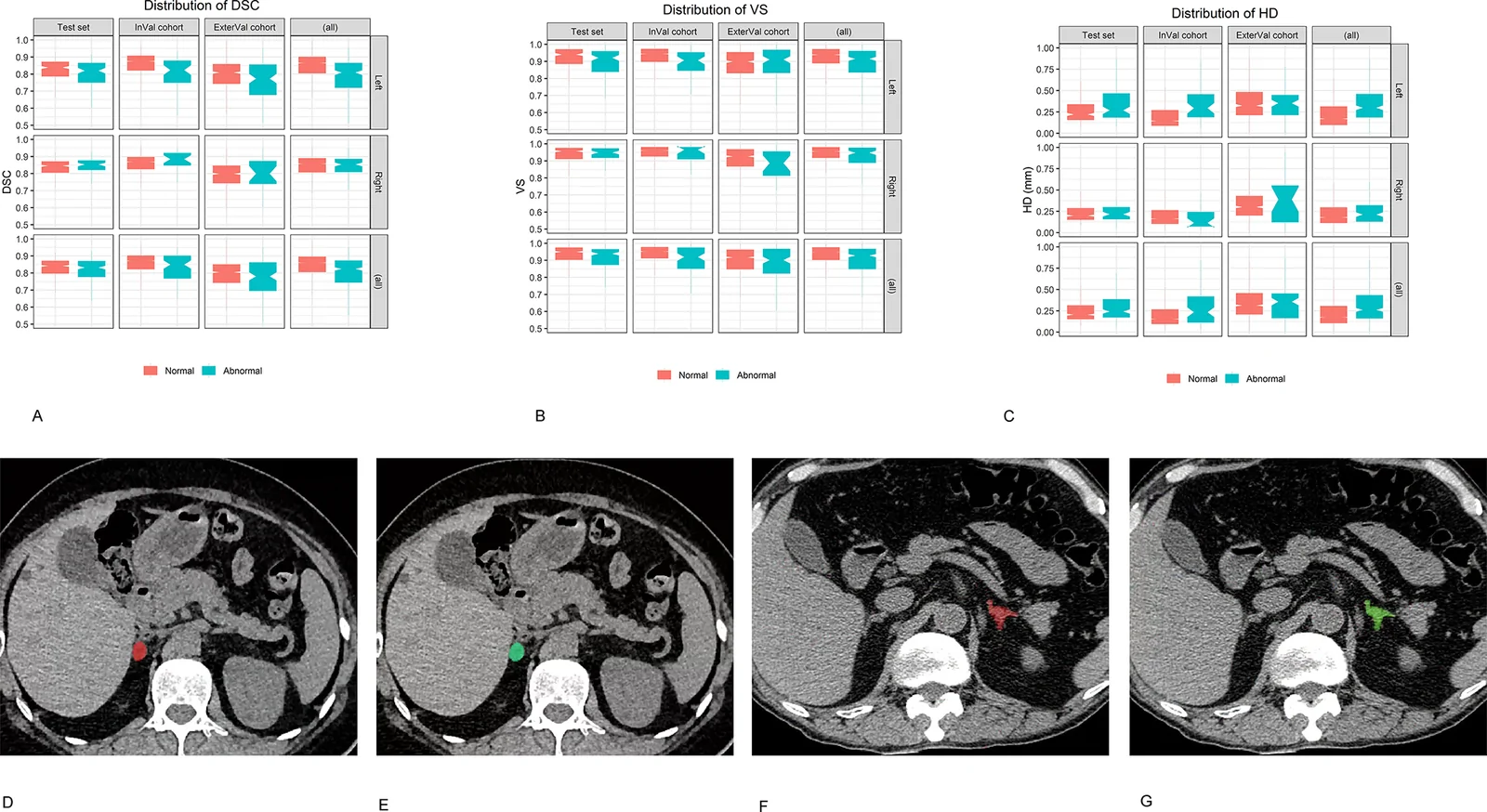
Evaluation metrics for the segmentation AI model. (**A**) displays the DSC results for the test set, internal validation cohort, and external validation cohort. The first row presents the DSC values for the left adrenal gland, which were categorized into normal and abnormal groups. The second row provides the DSC values for the right adrenal gland, using the same categorization. The third row shows the DSC values for all adrenal glands. The first column lists the DSC values in the test set for the left and right adrenal glands. The second column details the DSC values in the internal validation cohort. The third column presents the DSC values for all adrenal glands. (**B**) illustrates the volume similarity (VS), and (**C**) presents the average Hausdorff distance (HD) statistics. (**D**) and (**E**) present a concordant case of right adrenal adenoma. (**D**) displays the radiologist's manual segmentation of the right adrenal gland (red mask). (**E**) shows the corresponding segmentation predicted by the AI model (green mask). In this concordant case, the classification AI model correctly classified the adrenal gland as abnormal. (**F**) and (**G**) present a discordant case of left adrenal enlargement. (**F**) displays the radiologist's manual segmentation of the left adrenal gland (red mask). (**G**) shows the corresponding segmentation predicted by the AI model (green mask). This case represents a discordant classification result: the radiologists classified the left adrenal gland as abnormal due to its locally enlarged contour, whereas the classification AI model classified it as normal, likely attributable to the mild morphological changes. InVal, internal validation. ExterVal, external validation. DSC, Dice similarity coefficient. VS, volume similarity. HD, Hausdorff distance

The VS values for these groups were 0.947 (IQR: 0.893–0.973), 0.952 (IQR: 0.910–0.978), and 0.914 (IQR: 0.840–0.963), respectively. The HD values were 0.226 mm (IQR: 0.158–0.374), 0.163 mm (IQR: 0.0989–0.302), and 0.354 mm (IQR: 0.223–0.616), respectively. Subgroup analyses of the AI model’s segmentation performance (DSC, VS, and HD) stratified by adrenal gland status (normal vs. abnormal) and laterality (left vs. right) were also performed. Details are shown in Table 3 and illustrated in Fig. 4. 

**Table 3:** Evaluation metrics of the segmentation AI model [median (IQR)]

	Number of Adrenal Gland	DSC	VS	HD (mm)
**Test set**				
**Overall**	721	0.839 (0.783–0.871)	0.947 (0.893–0.973)	0.226 (0.158–0.374)
**Diagnosis**				
Normal	519	0.842 (0.789–0.871)	0.950 (0.903–0.975)	0.216 (0.155–0.345)
Abnormal	202	0.840 (0.775–0.873)	0.938 (0.859–0.966)	0.256 (0.179–0.493)
**Side**				
Right	361	0.846 (0.807–0.872)	0.956 (0.913–0.976)	0.203 (0.152–0.314)
Left	360	0.829 (0.754–0.867)	0.934 (0.873–0.966)	0.253 (0.167–0.460)
**Internal validation cohort**				
**Overall**	7325	0.870 (0.819–0.902)	0.952 (0.910–0.978)	0.163 (0.099–0.302)
**Diagnosis**				
Normal	7247	0.870 (0.819–0.902)	0.952 (0.911–0.978)	0.163 (0.099-0.300)
Abnormal	78	0.848 (0.758-0.900)	0.920 (0.851–0.975)	0.241 (0.116–0.460)
**Side**				
Right	3667	0.867 (0.823–0.897)	0.960 (0.926–0.981)	0.167 (0.106–0.281)
Left	3658	0.873 (0.813–0.907)	0.942 (0.894–0.973)	0.159 (0.092–0.328)
**External validation cohort**				
**Overall**	1545	0.799 (0.729–0.849)	0.914 (0.840–0.963)	0.354 (0.223–0.616)
**Diagnosis**				
Normal	1407	0.801 (0.738–0.849)	0.915 (0.843–0.962)	0.348 (0.221–0.608)
Abnormal	138	0.764 (0.643–0.850)	0.894 (0.817–0.967)	0.417 (0.244–0.682)
**Side**				
Right	773	0.795 (0.737–0.845)	0.925 (0.863–0.966)	0.342 (0.218–0.589)
Left	772	0.804 (0.721–0.854)	0.897 (0.822–0.957)	0.372 (0.231–0.643)

DSC: Dice similarity coefficient. VS: volume similarity. HD: average Hausdorff distance

### Results of the adrenal classification model

**Fig. 5:**
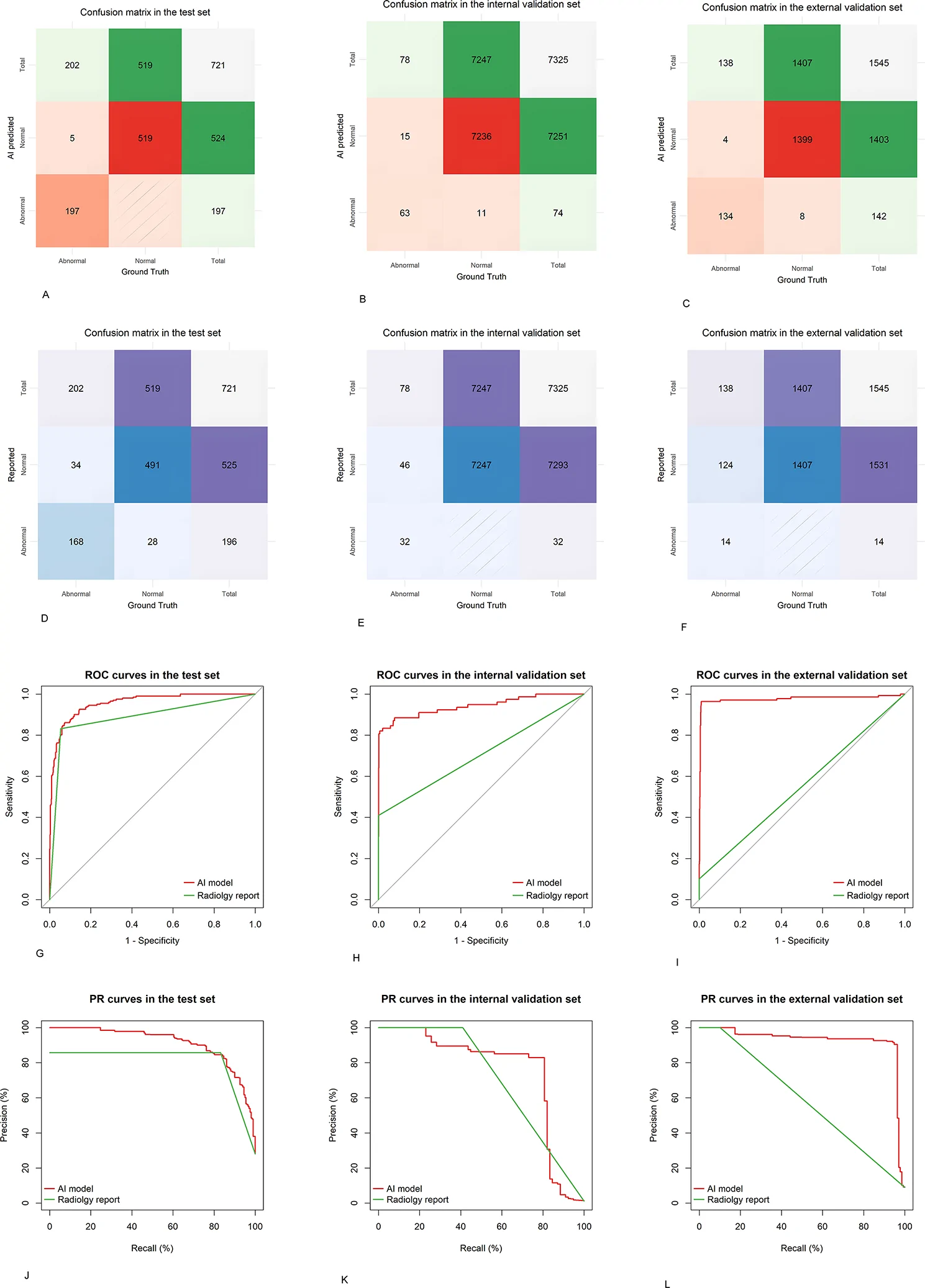
Confusion matrices, ROC curves and PR curves. (**A**)-(**C**) display the confusion matrices for the AI model across the test set, internal validation cohort, and external validation cohort. (**D**)-(**F**) show the confusion matrices for radiology reports across the test set, internal validation cohort, and external validation cohort. (**G**)-(**I**) present the ROC curves for both the AI model and radiology reports across the test set, internal validation cohort, and external validation cohort. (**J**)-(**L**) illustrate the PR curves for both the AI model and radiology reports across the test set, internal validation cohort, and external validation cohort. ROC, receiver operating characteristic. PR, precision-recall

The confusion matrices, PR curves, and ROC curves for the AI model and radiology reports for classifying adrenal glands across the test set, internal validation cohort, and external validation cohort are presented in Fig. 5. Details of the AUROC values, accuracy, sensitivity, specificity, positive predictive value, negative predictive value, positive likelihood ratio, and negative likelihood ratio for classifications in the three datasets are shown in Table 4. The DeLong test was used to compare the AUROC values between the AI model and routine radiology reporting across the datasets, and the results are shown in Table 5. 

**Table 4:** Evaluation metrics of the classification AI model [values (95% confidence intervals)]

	AUROC	ACC	SEN	SPE	PPV	NPV	PLR	NLR
**AI model**								
Test set	0.956 (0.941, 0.972)	0.908 (0.908, 0.909)	0.861 (0.814, 0.909)	0.927 (0.904, 0.949)	0.821 (0.769, 0.872)	0.945 (0.925, 0.965)	11.765 (8.620, 16.057)	0.150 (0.106, 0.211)
Internal validation cohort	0.942 (0.906, 0.978)	0.992 (0.992, 0.992)	0.821 (0.735, 0.906)	0.994 (0.992, 0.995)	0.582 (0.490, 0.674)	0.998 (0.997, 0.999)	129.266 (95.171, 175.576)	0.181 (0.112, 0.290)
External validation cohort	0.977 (0.956, 0.997)	0.988 (0.988, 0.988)	0.964 (0.933, 0.995)	0.990 (0.985, 0.995)	0.905 (0.857, 0.952)	0.996 (0.993, 1.000)	96.859 (57.457, 163.281)	0.037 (0.015, 0.087)
**Routing Radiology reporting**								
Test set	0.889 (0.861, 0.916)	0.914 (0.914, 0.914)	0.832 (0.780, 0.883)	0.946 (0.927, 0.965)	0.857 (0.808, 0.906)	0.935 (0.914, 0.956)	15.416 (10.695, 22.220)	0.178 (0.131, 0.242)
Internal validation cohort	0.705 (0.650, 0.760)	0.994 (0.994, 0.994)	0.410 (0.301, 0.519)	1.000 (1.000, 1.000)	1.000 (1.000, 1.000)	0.994 (0.992, 0.996)	Inf (NaN, Inf)	0.590 (0.490, 0.710)
External validation cohort	0.551 (0.525, 0.576)	0.920 (0.920, 0.920)	0.101 (0.051, 0.152)	1.000 (1.000, 1.000)	1.000 (1.000, 1.000)	0.919 (0.905, 0.933)	Inf (NaN, Inf)	0.899 (0.850, 0.950)

AUROC: area under the receiver operation characteristic curve. ACC: accuracy. SEN: sensitivity. SPE: specificity. PPV: positive predictive value. NPV: negative predictive value. PLR: positive likelihood ratio. NLR: negative likelihood ratio

**Table 5:** Comparison of the AUROCs for the AI model and routine radiology reporting across different datasets (DeLong test, *P* values)

	AI model			Routine Radiology reporting		
	Test set	Internal validation cohort	External validation cohort	Test set	Internal validation cohort	External validation cohort
**AI model**						
Test set	NA	0.482	0.115	< 0.001	< 0.001	< 0.001
Internal validation cohort	0.482	NA	0.104	0.021	< 0.001	< 0.001
External validation cohort	0.115	0.104	NA	< 0.001	< 0.001	< 0.001
**Routine radiology reporting**						
Test set	< 0.001	0.021	< 0.001	NA	< 0.001	< 0.001
Internal validation cohort	< 0.001	< 0.001	< 0.001	< 0.001	NA	< 0.001
External validation cohort	< 0.001	< 0.001	< 0.001	< 0.001	< 0.001	NA

Given the severe imbalance in the number of normal and abnormal adrenal glands in the internal and external validation cohorts, the PR curve was utilized to evaluate the classification capabilities of the AI model and routine radiology reporting. The respective AUPRC values for the AI model and radiology reporting in the test set were 0.913 and 0.809, those in the internal validation cohort were 0.753 and 0.708, and those in the external validation cohort were 0.927 and 0.591, respectively, with all differences being statistically significant (*P* < 0.05).

The AUROC values for the AI model in the test set, internal validation cohort, and external validation cohort were 0.956 (95% CI: 0.941–0.972), 0.942 (95% CI: 0.906–0.978), and 0.977 (95% CI: 0.956–0.997), respectively. The AUROC values for the routine radiology reporting across the three datasets were 0.889 (95% CI: 0.861–0.916), 0.705 (95% CI: 0.650–0.760), and 0.551 (95% CI: 0.525–0.576). The differences in AUROC values for the AI model across the three datasets were not statistically significant (all *P* > 0.05). In contrast, the differences in AUROC values for the routine radiology reporting across the three datasets were statistically significant (all *P* < 0.05). The AUROC values of the AI model were significantly greater than those of the routine radiology reporting in all three datasets (all *P* < 0.05).

## Discussion

### Main outcomes of this study

LDCT has been proven to be beneficial for lung cancer screening, with an increasing number of people undergoing annual LDCT examinations [31]. Consequently, radiologists have to interpret a growing volume of LDCT scans in their daily practice and they typically focus on pulmonary abnormalities in LDCT scans, often neglecting other unexpected pathologies within the scan range. These are known as incidental findings, which are serendipitously diagnosed in an asymptomatic or symptomatic patient undergoing imaging for an unrelated condition [32]. While not all of these incidental findings have significant clinical implications, some of them are indeed meaningful [33]. Some require immediate action, and some necessitate follow-up. It is worth mentioning all incidental findings in the report so as to facilitate clinical decision-making by clinicians [34, 35].

Studies have reported that adrenal abnormalities are prevalent but often neglected during radiology reporting, which highlights the need to raise awareness in the radiological community [6, 15, 16, 35]. Several factors may explain the observed discrepancy between AI performance and routine radiology reporting in our study. First, the radiology reports were generated during routine clinical workflow for thoracic LDCT examinations, where the primary clinical focus is pulmonary pathology assessment and lung cancer screening. In this context, adrenal findings may be intentionally omitted if considered clinically insignificant, or may not be systematically documented when the interpreting radiologist’s attention is directed toward the primary indication. Furthermore, prior studies have demonstrated that subspecialty expertise significantly influences the detection and management recommendations for incidental findings outside the radiologist’s primary area of focus [36, 37]. The interpreting radiologists in our cohorts were primarily thoracic or general radiologists rather than dedicated abdominal or uroradiology subspecialists. Therefore, the lower AUROC values observed for radiology reports (0.551–0.889) reflect routine reporting practices and workflow priorities rather than the intrinsic diagnostic capability of radiologists performing a focused adrenal assessment.

It is important to emphasize that our AI model is not intended to replace radiologist expertise, but rather to serve as an automated screening tool that flags adrenal abnormalities for subsequent review. The envisioned application is one of workflow augmentation: the AI model acts as a first reader to highlight adrenal regions requiring attention, thereby assisting radiologists, particularly those interpreting studies outside their subspecialty focus, in ensuring comprehensive evaluation without disrupting clinical efficiency.

### Segmentation efficacy of our AI model

Recent advancements in deep learning have led to impressive achievements in the precise delineation of abdominal organs within medical imaging. However, automatically segmenting the adrenal gland remains challenging due to its small size, indistinct borders from adjacent tissues, and significant variations in its shape and location. Koyuncu and colleagues [38] applied a blend of methods to segment the adrenal glands in 32 abdominal CT scans, obtaining a DSC of 0.83. Kim et al. [39] employed a U-Net architecture for adrenal segmentation across 308 abdominal CT scans, yielding a DSC of 0.701. Luo et al. [40] introduced a two-tiered approach to adrenal segmentation, achieving a DSC of 0.87 for 348 abdominal CT scans. Ahn et al. developed a two-stage deep learning model for adrenal nodule detection on conventional CT images, demonstrating robust performance in identifying focal adrenal lesions [41]. Similarly, Kim et al. proposed an automated deep learning framework for the detection and measurement of adrenal masses specifically in contrast-enhanced abdominal CT, achieving high accuracy in mass characterization and volumetric assessment [42].

Unlike these studies, which focused on abdominal CT images, our research centered on adrenal segmentation within thoracic LDCT images, which typically have lower image quality, thereby increasing the difficulty of our task. With such challenge, our study obtained competitive DSC values ranging from 0.799 to 0.870 across 9,606 adrenal glands in various validation sets, aligning with outcomes from prior studies. Our findings indicate that the DSC values for normal adrenal glands, ranging from 0.801 to 0.870, surpassed those for abnormal glands, which were between 0.764 and 0.848, with statistical significance (*P* < 0.05). This disparity may stem from an underrepresentation of abnormal adrenal cases in our training dataset. To bolster the efficacy of our segmentation model, future iterations should incorporate a more substantial dataset of abnormal adrenal glands.

### Classification efficacy of our AI model

Saiprasad et al. [22] employed a random forest classification framework for the automated segmentation of adrenal glands, followed by histogram analysis to detect abnormalities. Their combined approach yielded a sensitivity of 80% and specificity of 90% when 20 adrenal glands from abdominal CT datasets were examined for abnormalities. However, their study did not specify the exact conditions of adrenal abnormalities. Kim et al. [39] categorized adrenal glands as normal or hyperplastic on abdominal CT scans utilizing multilayer perceptron, support vector classification, a random forest classifier, and a decision tree classifier. These models relied on adrenal volume and anthropometric parameters such as height, weight, and sex. The sensitivity of the hyperplasia classification models ranged from 0.750 to 0.813, while the specificity ranged from 0.973 to 1.000 across 308 abdominal CT scans. Robinson-Weiss et al. [3] used an AI model to distinguish between normal and mass-containing adrenals on enhanced abdominal CT images, achieving a sensitivity of 69% (95% CI: 58, 79) and a specificity of 91% (95% CI: 90, 92) in a test set of 991 cases.

Our study differed from the aforementioned ones in three main aspects. First, we focused on images from thoracic LDCT scans. Second, we utilized a larger dataset during training, conducted a broader range of validations, and compared our AI model results with those of routine radiology reporting, demonstrating the potential clinical benefits of using AI model. Our validation cohorts included an internal validation cohort (7325 adrenal glands) and an external validation cohort (1545 adrenal glands). The model showed robust classification performance in both cohorts, with sensitivity ranging from 0.821 to 0.964, specificity ranging from 0.927 to 0.994, ROC AUC ranging from 0.942 to 0.977, and PR AUC ranging from 0.753 to 0.927. The AI model significantly outperformed radiology reports in detecting adrenal abnormalities in both the internal and external validation cohorts. Third, our study classified adrenal glands into two categories: normal and abnormal, rather than the normal vs. mass-containing adrenal [3] or normal vs. Hyperplasia [39] classification used in previous studies. Our definition of abnormalities encompasses various types of morphological and density abnormalities of the adrenal glands, aligning more closely with clinical reality.

In our study, the rate of adrenal abnormalities was 1.8% (65 cases out of 3681) in the internal validation cohort and notably higher at 15.4% (127 cases out of 779) in the external validation cohort. This significant difference in incidence rates can likely be attributed to the demographic differences between the cohorts, with the external cohort having an older patient population (median age 60.0 years, IQR: 48.0–69.0) than the internal cohort (median age 47.0 years, IQR: 37.0–60.0), a disparity that was statistically significant (*P* < 0.001). Age is a known affective factor for the occurrence of adrenal abnormalities, as evidenced by studies showing an increase in prevalence with age, from as low as 1% in individuals under 30 years to 7–10% in those aged 70 or older, with the highest rates observed in the 5th to 7th decades of life [32]. Furthermore, the reported rates of adrenal abnormalities in the literature exhibit considerable variation, which can be attributed to different criteria for identifying adrenal masses. A comprehensive review of the literature suggests a prevalence of adrenal masses ranging from 1.0% to 8.7% in autopsy studies and 0.5% to 2% in abdominal CT scans, with the likelihood of adrenal enlargement ranging between 0% and 21.33% [15]. This variability underscores the need for a standardized approach to defining adrenal abnormalities. Our proposed AI model is designed to detect these adrenal abnormalities accurately. Its application in future large-sample studies could enhance our understanding of adrenal abnormality incidence and potentially serve as an initial screening tool in community health assessments, offering a valuable contribution to the field.

### Limitations of this study

This study was limited in three aspects. First, the classification AI model we proposed only made a binary classification judgment of the adrenal glands as either normal or abnormal without further refining the classification for abnormal cases. This is due to the fact that the current AI model is designed to act as the first reader, like serving a triage function. This AI model is intended to sensitively detect suspicious adrenal abnormalities, providing a prompt to radiologists, who then make final diagnosis of the specific abnormality. The segmentation model employed standard Dice loss, which treats false negatives and false positives equally. Given the slightly lower DSC observed for abnormal glands compared to normal glands, alternative loss functions such as Tversky loss or Focal-Tversky loss may improve segmentation recall for the minority abnormal class. Future iterations of the model should evaluate whether such loss functions can enhance both segmentation quality and downstream classification performance. Second, this research was not conducted in conjunction with clinical manifestations and follow-up examination results to clarify the clinical significance of adrenal imaging abnormalities. Incidental enlarged adrenal glands are often neglected during the reporting of abdominal CT, partly due to their unknown clinical significance and partly due to their unclear further management. This requires not only the input of CT images but also the integration of patients’ clinical information and outcome data to develop a clinical predictive model. Third, this study only tested the model on chest LDCT images that from two hospitals. In practice, there are also application scenarios in routine thoracic and abdominal CT diagnosis. Our AI model was trained on a combination of LDCT and standard-dose abdominal CT, without explicit domain alignment techniques such as intensity normalization or dose simulation. Consequently, the model’s generalizability to other CT protocols remains to be validated. Future testing and, if necessary, iteration of the AI model should extend its application to all chest and abdominal CT screening, thus providing radiologists with more comprehensive assistance.

## Conclusions

The deep learning models showed promise in automated adrenal segmentation and classification, highlighting AI’s potential to improve detection of adrenal incidental abnormalities in LDCT scans.

## Data Availability

The datasets used and/or analysed during the current study are available from the corresponding author on reasonable request.

## Abbreviations

LDCT: Low-dose computed tomography
DSC: Dice similarity coefficient
VS: Volume similarity
HD: Average Hausdorff distance
ROC: Receiver operating characteristic
PR: Precision-recall
IQR: Interquartile range
